# Occurrence of Cervical Spine Pain and Its Intensity in Young People with Temporomandibular Disorders

**DOI:** 10.3390/jcm13071941

**Published:** 2024-03-27

**Authors:** Martyna Odzimek, Waldemar Brola

**Affiliations:** 1 Doctoral School, The Jan Kochanowski University, Żeromskiego 5, 25-369 Kielce, Poland; 2 Institute of Health Sciences, Collegium Medicum, The Jan Kochanowski University, Al. IX Wieków Kielc 19A, 25-516 Kielce, Poland; wbrola@wp.pl

**Keywords:** cervical pain, temporomandibular joint (TMJ), temporomandibular disorders (TMDs)

## Abstract

**Background**: The main aim of this cross-sectional study was to compare the occurrence and severity of cervical spine pain in young adults diagnosed with TMDs with a healthy control group (without TMDs). **Methods**: The study was conducted from June to July 2023. Inclusion criteria were age (18–30 years), cervical spine pain (for at least 1 month), and consent to participate in the study. The study was conducted based on RDC/TMD protocol, an original questionnaire, and a physiotherapeutic examination focused on detecting TMDs. The cervical pain level was assessed using the Visual Analogue Scale (VAS). Thus, a total of 95 subjects were registered for the trials, 51 people (53.7%) constituted the control group (without TMDs), while 44 (46.3%) people constituted the study group (with TMDs). **Results**: The mean age of people participating in the study was 22.2 ± 2.2 years in the study group and 22.5 ± 3.1 years in the control group. The largest group was people aged 21–25 (*n* = 51 people, 53.7%). Patients from the study group more often experienced pain in the stomatognathic system during palpation (both in the muscle, joint, and musculoskeletal groups) and had reduced mobility of the temporomandibular joints in every movement (*p* < 0.001). People from the study group were also characterized by less mobility of the cervical spine (*p* < 0.05), apart from extension movement (*p* > 0.05). The analysis showed that of the 95 people participating in the study, 85.4% reported problems in the cervical spine area (*n* = 81), of which almost all people in the study group struggled with this problem (*n* = 43, 97.7%). It was found that cervical spine pain was significantly more common in people with TMDs (*p* < 0.05, chi^2^ = 10.118, df = 1, r_c_ = 0.31). The level of pain was significantly higher in people from the study group (*p* < 0.001, chi^2^ = 45.765, df = 4, r_c_ = 0.57). **Conclusions**: Our research has shown that the occurrence of cervical spine pain is more common in the group of young people with temporomandibular disorders (TMDs). In young people, this problem is rarely recognized and properly treated.

## 1. Introduction

Temporomandibular disorder (TMD) is a term used to describe structural and/or functional disorders within the masticatory musculoskeletal system. They may involve joints as well as muscle or ligament structures [1,2,3,4]. Due to its complex etiology and the influence of many external factors, it is considered the second most common musculoskeletal pain [5,6,7]. The dominant causes of TMDs include psychosocial disorders (stress, depression, mental illnesses, emotional disorders) [4,8,9], posture defects (changes in the spine, upper limbs, lower limbs, and pelvis) [5,6,7,8,9,10], genetic and/or developmental changes [11], or hormonal disorders [11]. The most common causes of TMDs are anatomical changes in the structure of the temporomandibular joints and adjacent structures, incorrect occlusion, previous injuries (including micro-injuries), parafunctions (occlusive, non-occlusive), or inflammation of the masticatory musculoskeletal system [5,6,7,10]. The main symptoms of TMDs are pain (muscle and/or articular), problems with chewing food, clicking in the joint, soreness in the joint, pain in the ears and tinnitus, changes in the appearance of the face (excessive masticatory hypertrophy), headache and/or neck pain, limitation in opening the mouth, decreased or increased mobility of the temporomandibular joints, rubbing or clenching of the teeth during the day and/or night [4,7,12,13,14,15]. A careful analysis of research results shows that the incidence of TMD in the world population is 34% [6]. When it comes to European areas, this problem affects up to 29% of the population, including the following statistics: under 18 years of age, it is 18%, in the age group 18–60, it is 41%, and in the age group over 60, it is 32% [6]. In the case of children and adolescents, this problem occurs in 40 to even 90% of people [4,5,6,7,8,9,10]. Such a large discrepancy in results may be due to the inclusion of different diagnostic criteria, different methodologies, sample size, or consideration of painful and non-painful symptoms of TMDs [6,7].

Cervical spine pain is a multifactorial disease and a health problem that affects up to 70% of the population, causing a decrease in their quality of life [15]. They are a significant socio-economic problem right after lumbar spine pain. In the vast majority of studies, it is difficult to clearly determine the cause of pain, which is a significant problem in introducing appropriate therapy [15]. According to a 2017 epidemiological study, the highest standardized incidence of cervical spine pain was recorded in East Asian regions (1029 cases per 100,000 inhabitants), while the lowest was recorded in Latin American regions (624 cases per 100,000 inhabitants). Disorders of the cervical spine were much more common in women (166 million) than in men (122.7 million). The analysis showed that the number of years lived with disability is higher in women than in men (10.0–25.1 vs. 7.4–18.9). In addition, it has been determined that the standardized age of neck pain incidence increases with age, up to 70–74 years, and then a decrease is noted [14]. The factors influencing cervical spine pain include psychological factors, emotional stress, anxiety disorders, depression, cognitive factors, sleep problems, socioeconomic problems, concomitant neuromusculoskeletal diseases, autoimmune diseases, and genetic factors [14].

In the scientific literature, there is information on the relationship between cervical spine pain and temporomandibular disorders, which may include biomechanical, neurophysiological, or neuroanatomical aspects [16]. The connection of the cervical spine with the temporomandibular joints shows that they have a direct impact on each other’s functioning. Abnormal tension within the locomotor system of the masticatory system may be related to the position of the head and cervical spine (tension within the paraspinal muscles). This is the overlapping between primary afferent neurons from either the trigeminal region or the trigemino-cervical complex, which have implications for pain referral [7,17].

The study aimed to verify the main hypothesis that cervical spine pain in young people may be related to TMDs. An attempt was made to verify the above hypothesis because young people do not yet have degenerative changes in the cervical spine, but they often experience pain in this section.

## 2. Materials and Methods

### 2.1. Ethics Statement and Information about Project

The study was approved by the Bioethics Committee of the Collegium Medicum of the Jan Kochanowski University (date of approval: 19 May 2023, No. 22/2023). Prior to the study, participants were informed about the objectives, methods, benefits, as well as risks of participating in the study. Each person gave informed, voluntary consent to participate in the study, which was documented on consent forms available for inspection by the first author. In addition, all participants in the study were covered by accident insurance (certificate No. COR401865). All procedures were carried out in accordance with the 1964 Declaration of Helsinki, as amended. The project was funded by the Jan Kochanowski University in Kielce.

### 2.2. Study Population

The eligibility criteria for potential study participants were age (18–30 years—the more frequent occurrence of TMDs symptoms in these people and the possibility of excluding degenerative changes in the study population), cervical spine pain experienced for at least 1 month, and voluntary consent to participate in the study. The exclusion criteria were age (under 18 or over 30 years of age), neurological diseases (in particular, stroke, multiple sclerosis, cancer, facial or trigeminal nerve palsy), traffic accident (within the last year), head or neck injuries (within the last year), head and/or neck surgeries, cervical spine pain lasting more than 12 months, regular use of analgesics and anti-inflammatory drugs, articular hypermobility, degenerative and/or rheumatoid diseases of the cervical spine, and lack of consent to participate in the study. This study was conducted at the Faculty of Collegium Medicum of the Jan Kochanowski University in Kielce (after obtaining the consent of the University authorities) and in a private physiotherapy practice in the period from June 2023 to July 2023. A random sample of 100 people aged 18–30 qualified for the study, of which 5 results were rejected due to formal deficiencies (lack of consent, lack of signature, incomplete documentation, incorrectly completed questionnaire). Among the 95 people participating in the study, 51 people (53.7%) made up the control group (no history of TMDs), while 44 (46.3%) made up the study group (with a history of TMDs).

### 2.3. Methods

Information about TMDs was collected using a questionnaire recommended by the European Academy of Craniomandibular Disorders (EACD). The screening protocol contains four questions [18]:

1. Do you feel pain when you open your mouth wide or chew (at least once a week or more)?
2. Do you have pain in your temple, face, temporomandibular joint or jaw (at least once a week or more)?
3. Do you feel like your jaw is blocked or that you have problem with open it wide?
4. Do you get headaches more than once a week?

On the basis of the study conducted using the European Academy of Craniomandibular Disorders (EACD) and the study based on the RDC/TMD [1] protocol (the part related to the clinical trial excluding examination of the location of the click and its elimination), a division into the study and control groups was made (a current diagnosis). In addition, the RDC/TMD clinical examination has been supplemented with an original part containing information on general health condition, palpation of neck muscles, and examination of myofascial trigger points. Only after this part did physiotherapist measure the mobility of the cervical spine and used the Visual Analog Scale (VAS) regarding back pain. Depending on the intensity of pain experienced, patients were divided into groups: 0—no pain; 1–3—mild pain; 4–7—moderate pain; 8–9—severe pain; 10—unbearable pain [19].

The range of motion of the jaw was examined using a Fanger electronic caliper (measurement error ±0.01), and the range of motion in the cervical spine was examined using a Baseline inclinometer (product code: 4372-4405E, measurement error ±0.5°). The clinical examination was conducted by a physiotherapist specialized in the treatment of temporomandibular dysfunction under medical supervision (the dentist and the neurologist). A detailed course of the study is presented in Table 1. All data were collected during a single visit, performed by the same person under standardized conditions, and devices and methods of confirmed scientific significance were used for the study [20,21].

### 2.4. Statistical Analysis

Statistical analysis was performed using Statistica™ version 13.3 (TIBCO Software Inc., Palo Alto, CA, USA) and in Microsoft Excel. G*power software version 3.1.9.7 (Düsseldorf, Germany) was used for the statistical power of the sample size, and the total number of participants was calculated to be 77 (effect size = 0.9, α-error probability = 0.05, power = 0.9). Thus, a total of 95 subjects were registered for the trials. Statistical description techniques and the Shapiro–Wilk test of normality were applied for descriptions of the groups and the variables. The parametric Student’s *t*-test was applied because the variables are normally distributed.

To verify the secondary hypotheses, the same method was applied for the comparison of quantitative variables (e.g., age) between the groups, and a contingency table analysis with chi-square analysis was performed to compare the groups in terms of nominal and ordinal variables. The magnitude and clinical relevance of the scores were evaluated based on effect size statistics. According to Cohen’s benchmarks, a value of 0 to 0.20 denotes a negligible effect size, a value of 0.21 to 0.5 denotes a small effect size, a value of 0.51 to 0.80 denotes a medium effect size, and a value >0.80 denotes a large effect size. *p*-values < 0.05 were considered statistically significant.

## 3. Results

A total of 95 people took part in the study, of which 51 were included in the control group (*n* = 38 women, 74.5% vs. *n* = 13 men, 25.5%), and 44 people were included in the study group (*n* = 37 women, 84.1% vs. *n* = 7 men, 15.9%). The mean age of the participants was 22.4 years (±2.7), and there were no significant differences between the study group (22.2 ± 2.2) and the control group (22.5 ± 3.1). In addition, respondents had to answer a question about their health. In both the study group and the control group, no one described their health as poor or satisfactory (*n* = 0, 0.0%, respectively). Most people described their health as good (*n* = 49, 51.6%), including 22 people from the control group (43.1%) and 27 people from the study group (61.4%). A total of 34 respondents (35.8%), including 20 people from the control group (39.2%) and 14 people from the study group (31.8%), described their health as very good. Only 12.8% of the respondents (*n* = 12) described their health as excellent, and the vast majority of them were from the control group (*n* = 9, 17.7%), compared to the study group (*n* = 3, 6.8%). Detailed characteristics of the group are presented in Table 2.

In the next part of the study, respondents answered questions about the occurrence of symptoms within the masticatory system. The majority of patients in the study reported headaches (*n* = 54, 58.8%), of which the vast majority were controls (*n* = 43, 95.5%). Only two people had not noticed this problem in recent times (*n* = 2, 4.5%). In addition, the respondents often indicated pain in the masticatory muscles and/or neck (*n* = 44, 46.3%), and in the vast majority of cases, this problem occurred in the study group (*n* = 37, 84.1%) rather than in the control group (*n* = 7, 13.7%). In addition, pain or discomfort in the temporomandibular joint area was frequently reported (*n* = 41, 43.2%), where again the vast majority were patients from the study group (*n* = 38, 86.4%), compared to only three cases in the control group (*n* = 3, 5.9%). Clenching of teeth, tinnitus, and crackling in the joint were very common symptoms in the study group. They constituted 56.8% (*n* = 25), 43.2% (*n* = 19), and 45.5% (*n* = 20) of this group, respectively, compared to a significantly lower occurrence in the control group (*n* = 13, 25.5%, *n* = 12, 23.5%, *n* = 7, 13.7%, respectively). The respondents were least likely to report the problem of increased or decreased mobility of the temporomandibular joints (*n* = 17, 17.9%) and numbness in the mandible (*n* = 6, 6.3%). The above problems were definitely more often noticed by the study group (*n* = 17, 38.6% and *n* = 5, 11.4%, respectively) than by the control group (*n* = 1, 2.0% and *n* = 1, 2.0%, respectively). The results of this study showed significant associations, with effect sizes in the range of 0.42–0.94 for Cohen’s values, between pain and/or discomfort in the TMJ area (ES = 0.86, *p* < 0.001), pain in the chewing muscles (ES = 0.68, *p* < 0.001), clicking or snapping sound in the TMJ (ES = 0.94, *p* < 0.001), increased or decreased mobility of the TMJ (ES = 0.92, *p* < 0.001), rubbing and/or clenching of teeth and headaches (ES = 0.94, *p* < 0.001). Detailed figures and statistical analysis are presented in Table 3.

The next part of the study was based on detailed palpation in both the study group and the control group. In each of the groups, it was possible to choose more than one option (multiple choice). During palpation of the masticatory muscles and temporomandibular joints, 57 respondents (60.0%) reported no pain in the above locations. The vast majority of this group were subjects from the control group (*n* = 40, 78.4%) rather than from the study group (*n* = 17, 38.6%). Patients in the control group were most often diagnosed with problems with muscle pain on the left side of the face (*n* = 7, 13.7%). Problems in the form of pain in the left side of the joint (*n* = 1, 2.0%), simultaneous pain in the left side of the joint (*n* = 2, 3.9%), muscle pain on the right side (*n* = 2, 3.9%), pain in the right side (*n* = 2, 3.9%), or simultaneous pain in the muscle and joint on the right side (*n* = 1, 2.0%) were reported sporadically. Patients from the study group reported symptoms during palpation in the form of muscle pain on the left (*n* = 19, 43.2%) and/or right (*n* = 12, 27.3%) sides of the face or simultaneous muscle and joint pain on the left and/or right side (*n* = 10, 22.7% and *n* = 10, 22.7%, respectively). Less frequently, pain problems were observed only in the left (*n* = 6, 13.6%) or right (*n* = 5, 11.4%) temporomandibular joint. The results of this study showed significant associations, with effect sizes in the range of 0.28–0.98 for Cohen’s values, between without facial pain (ES = 0.76, *p* < 0.05), muscle pain on the left side (ES = 0.68, *p* < 0.05), muscle pain on the right side (ES = 0.74, *p* < 0.05), and muscle and joint pain on the right side (ES = 0.98, *p* < 0.001) (Table 4).

In the next part of the study, detailed measurements of the range of motion of the temporomandibular joints were made with the use of electronic calipers. In the control group, significantly higher results were recorded in terms of performed movements. Mandibular abduction in the control group averaged 42.3 ± 14.8 mm, and pain during movement was reported by only one person (2.0%). In the control group, the range of this movement was significantly lower (30.9 ± 8.2 mm), and pain during movement was noted in 19 people (43.2%). Protrusion movement was performed to a greater extent in the control group (8.2 ± 2.1 mm) than in the study group (4.6 ± 1.9 mm), and pain during movement was reported by two people (3.9%) from the first group and as many as twenty-seven people (61.4%) from the study group, respectively. Similar associations are visible when performing lateral movements. In the control group, lateral movement to the left and right was in a similar range (9.2 ± 1.7 mm and 9.8 ± 1.1 mm, respectively), and pain was reported by only one person (2.0%) during lateral movement to the right. In the study group, lateral movement to both the left and right was found to a lesser extent (8.3 ± 1.3 mm and 7.8 ± 1.8 mm, respectively). Pain during lateral movement to the left was reported by 12 people (27.3%) and by 10 people (22.7%) to the right. The results of this study showed significant associations, with effect sizes in the range of 0.59–0.95 for Cohen’s values, between abduction (ES = 0.95, *p* < 0.001), protrusion (ES = 0.79, *p* < 0.001), lateral movement to the left (ES = 0.59, *p* < 0.001), and lateral movement to the right (ES = 0.64, *p* < 0.001) (Table 4). Figures and statistics are presented in Table 5.

The last part of the study was the measurement of the range of motion of the cervical spine using a Baseline inclinometer. Significantly better results were recorded in the respondents from the control group than those from the study group. In the flexion and extension control group, the average range of motion was 45.6 ± 12.1° and 74.1 ± 5.6°, while in the study group, it was 40.1 ± 9.9° and 65.2 ± 6.3°, respectively. Cervical spine flexion pain was reported in one patient in the control group (2.0%) and ten patients in the study group (22.7%). Pain during extension was reported by one person (2.0%) from the control group and nine people from the study group (20.5%). Left rotation was better in both the control group (68.9 ± 8.2°) and the study group (61.7 ± 10.9°) compared with the slightly weak right-hand rotation (67.5 ± 12.7° in the control group, 59.1 ± 11.2° in the study group). Pain during right rotation was reported by two subjects (3.9%) from the control group and eight subjects (18.2%) from the study group. On the other hand, during the left rotation, no person from the control group reported abnormalities, while in the study group, it was eight respondents (18.2%). In the case of lateral flexion movement to the right and left sides, identical results were noted in the study group (mean 45.3 ± 7.9°, 45.3 ± 7.4°), and pain during movement was reported by five people (11.4) and six people (13.6), respectively. Better results were presented by people from the control group, where the lateral flexion movement to the right side was on average 50.7 ± 8.7°, and to the left side 52.5 ± 8.5°. Pain was reported by three people (5.9%) and one person (2.0%), respectively. The results of this study showed significant associations, with effect sizes in the range of 0.31–0.90 for Cohen’s values, between flexion (ES = 0.49, *p* < 0.05), rotation to the right (ES = 0.70, *p* < 0.05), rotation to the left (ES = 0.75, *p* < 0.05), side bend to the right (ES = 0.64, *p* < 0.05), and side bend to the left (ES = 0.90, *p* < 0.05) (Table 4). The data are shown in Table 6.

In the last part of the survey, the respondents answered about their cervical spine pain and the level of its intensity. A total of 85.3% of the participants (*n* = 81) in the study reported cervical spine pain in the past month. The absence of upper back disorders was noted in 14.7% (*n* = 14) of the respondents. Problems were much more common in the study group (*n* = 43, 97.7%) than in the control group (*n* = 38, 74.5%). The cervical pain was not reported by only one person (2.3%) in the study group and thirteen people (25.5%) in the control group. The intensity of cervical spine pain was assessed using the Visual Analogue Scale (VAS). The mean level of pain intensity in the control group was 1.6 ± 0.8, while in the study group, it was 4.7 ± 3.2 (*p* < 0.001). None of the respondents (both control and subject) described their pain as unbearable. Severe pain was noted only in the study group (*n* = 13, 29.5%), while it was reported by in none of the control group (*n* = 0, 0.0%). Moderate pain was more common in the study group (*n* = 20, 45.5%) than in the control group (*n* = 4, 7.8%). Mild pain was reported by the highest number of patients from the control group (*n* = 34, 66.7%) and slightly less from the study group (*n* = 10, 22.7%). The results of this study showed significant associations, with effect sizes in the range of 0.85–0.98 for Cohen’s values, between the cervical spine pain (ES = 0.85, *p* < 0.05) and intensity of pain in the cervical spine (ES = 0.98, *p* < 0.001), Detailed figures and statistics are given in Table 7.

## 4. Discussion

The main aim of this cross-sectional study was to compare the occurrence and severity of cervical spine pain in young adults diagnosed with TMDs with a control group (without TMDs). Patients from the study group more often experienced pain in the stomatognathic system during palpation (both in the muscle, joint, and musculoskeletal groups) and had reduced mobility of the temporomandibular joints in every movement (*p* < 0.001). People from the study group were also characterized by less mobility of the cervical spine (*p* < 0.05), apart from extension movement (*p* > 0.05). It was found that cervical spine pain was significantly more common in people with TMDs (*p* < 0.05, ES = 0.85). The level of pain was significantly higher in people from the study group (*p* < 0.001, ES = 0.98).

The analysis of the available scientific literature is not uniform in terms of the prevalence of this problem. It is estimated that this problem occurs in 3–34% of the population [6,22,23,24,25]. Polish scientific literature reports that this problem may occur in 26.5% [23] to even 48.9% [26] of the population living in these areas. The variety of diagnostic criteria, group size, age of study participants, and the presence of comorbidities may have a significant impact on epidemiological differences in the occurrence of temporomandibular disorders [21]. The examined literature shows that temporomandibular disorders occur much more often in women than in men (2:1, but sometimes in the range of 3–4:1) [10,13,26,27,28,29,30,31]. In the case of the authors of the above studies, as well as the study by Wolan-Nieroda and co-authors [32], only people from the age group of 18–30 were qualified for the analysis, which may slightly distort the view on the occurrence of particularly painful forms of TMDs. There is no information in the scientific literature on the subjective assessment of the health status of people with temporomandibular disorders. Our own research showed that people with temporomandibular disorders most often described their health condition as good (61.4%) or very good (31.8%), and least often as excellent (6.8%). Similar results were obtained in the control group (43.1%, 39.2%, and 7.7%, respectively). None of the people participating in the study described their health condition as poor or sufficient.

Disorders of the masticatory system may affect both the muscular and/or osteoarticular systems. The most common symptoms of diseases of the stomatognathic system are pain in the muscles of the masticatory system [4,7,10], headaches (21.7–56.5%) [33,34,35,36], and clicking in the temporomandibular joints (26.7%) [33]. People with masticatory system disorders report migraine headache problems more often than people without TMD [36]. Similar study results were presented by the authors of the above article, where headaches were reported more often by people in the study group (95.5%) than in the control group (23.5%), and the result was statistically significant (*p* < 0.001). Moreover, the scientific literature indicates that headaches affect women more often than men (1.7:1) [37]. Improper tension of the muscles of the masticatory system and/or neck may have a significant impact on the occurrence of headaches. It is estimated that this pathology occurs in 32.2% [35] to even 97.3% [38,39] of people with TMD. Our own research also confirms the above relationship (*p* < 0.001; control group: 13.7%; study group: 84.1%). Another important aspect analyzed in research is pain or discomfort in the area of the temporomandibular joint, which occurs in as many as 36% of people [35]. This relationship was also demonstrated in our own research (*p* < 0.001, 86.4% of respondents). It is worth mentioning that the potential relationship between temporomandibular disorders and bruxism is often discussed in the scientific literature. According to researchers, there is a relationship between these variables [40], and bruxism is found in 17.9% [41] to 96.6% of people with TMD [40].

Our study confirms the above theory (*p* < 0.001), but no such large differences in numbers and percentages were found (control group, *n* = 13, 25.5%; study group, *n* = 35, 56.8%). There is scientific evidence that tinnitus is more common in people with TMDs. It is estimated that this problem may affect from 11.4% [40] to even 60.7% [42,43]. However, other systematic reviews indicate that the prevalence of tinnitus in people with TMD may range from 3.7% to even 70% [44,45]. Our own research does not confirm statistical significance (*p* > 0.05), and the differences between the groups were not significant (23.5% in the control group vs. 43.2% in the study group). It is worth noting that in addition to all of the above symptoms, patients may also report a clicking in the TMJ. They may occur in 14.3% to as many as 52% of people with TMDs [42], which was also noticed by the authors of the above article (*p* < 0.001, control group: 13.7% vs. study group: 45.5%).

The results of the study obtained by the authors in the field of detailed physical examination within the musculoskeletal system of the masticatory organ and the cervical spine allow us to conclude that there are significant differences between the control group and the study group. Patients from the study group were characterized by more frequent pain during palpation (both in muscle, joint, and musculoskeletal groups). In addition, decreased mobility of the temporomandibular joints in each movement was noted (abduction, *p* < 0.001; protrusion, *p* < 0.001; lateral movement to the left, *p* < 0.001; lateral movement to the right, *p* < 0.001), as well as more frequent reporting of pain during their performance than in the control group. It is also worth noting that the subjects in the study group were characterized by lower mobility of the cervical spine, and the results obtained were statistically significant (*p* < 0.05), except for extension movement (*p* > 0.05). By focusing exclusively on young people, we have been able to eliminate many comorbidities that could significantly affect our research results. Similar studies, also conducted in the 18–30 age group, were conducted by Wolan-Nierod and co-authors [32], but on a smaller number of patients (control group, *n* = 30; study group, *n* = 30). The results obtained by them are similar to those obtained by the authors of the above work. People with TMDs achieved lower measurements of temporomandibular joint mobility and cervical spine. Statistically significant correlations concerned lateral flexion to the right or left and rotation to the right or left. In addition, there was a statistically significant reduction in TMJ mobility in each movement (*p* < 0.001). The analysis did not investigate the protrusion movement. Similar results were also obtained by Kitsoulis and co-authors, who emphasized that people with disorders within the masticatory motor system achieved lower scores when measuring TMJ mobility [46]. A study by Ferreira and co-authors showed that people with TMDs experienced reduced mobility of the cervical spine during flexion and extension movements, and poorer outcomes when performing the flexion and rotation test and the craniocervical flexion test (all *p* < 0.05). It is worth noting that the study was conducted only in a group of women, and the results correlated with pain and poor neck muscle function, which was also noted by the authors of the above studies [47]. In addition, researchers note that patients with TMDs are more likely to have segmental limitations or tender trigger points within the muscles than in control groups [48,49].

There is still no single position in the scientific literature on the influence of posture on the occurrence of TMDs [50]. It is estimated that spinal pain may occur in up to 48% of people with TMDs, and its cervicogenic origin is much higher in the group of people with TMDs [17]. Moreover, the research confirms that there is a relationship between painful TMDs and pain in the cervical spine [51] and a correlation between TMD and limited mobility and pain in the cervical spine [38]. The implementation of appropriate splint therapy may reduce pain in the cervical spine and increase mobility [30]. It is worth noting that incorrect position of the head (in protraction) may be a factor in the development of TMDs [52] and cause pain in the cervical spine or the TMJ [53]. In addition, muscle tension in the stomatognathic system may affect changes in postural control due to numerous neurological, proprioceptive, muscular, articular, or ligamentous connections [52]. In contrast to the above studies, the results of the analysis by Weber and co-authors, conducted in a group of women aged 19 to 35, stand in contrast to the above studies. Studies show that the coexistence of cervical spine symptoms and TMDs may be more related to the innervation of the trigeminocervical complex than to deviations in body posture [54,55]. Another team of researchers came to similar conclusions, determining that there is no relationship between the occurrence of symptoms of TMDs and curvatures of the cervical spine [56]. In addition, the results of the study do not support the thesis that body posture can exacerbate or cause symptoms of temporomandibular disorders. However, these results were conducted on small groups, which had a significant impact on the result obtained [57]. Significant attention is paid to the occurrence of pain in Pedroni syndrome [58], where some of the respondents at the time of assessment assessed the intensity of pain as mild (35.5%) or bothersome (21.42%). The authors of the above study also noted a higher intensity of pain in people with TMDs than in those in the control group (*p* < 0.001). Moreover, the latest research shows that a group of people with myopia and TMD was characterized by greater muscle tenderness, longer rest periods, and lower functional bioelectrical activity of muscles. Therefore, it can be concluded that the organ of vision is clinically related to the masticatory and cervical muscles. It is also worth adding that researchers have noticed that the thickness of the choroid in people with myopia is related to muscle tenderness. Additionally, TMDs and myopia worsen sleep quality, which shows how complex the problem it is [59,60,61]. An important role in the treatment of people with TMDs is the introduction of interdisciplinary therapy based on the cooperation of dentists, physiotherapists, speech therapists, or doctors of other specialties (orthopedics, laryngologists, or neurologists) [21,32]. A significant impact of appropriate treatment on the reduction in TMDs has been reported [25]. Properly conducted physiotherapy and dental treatment can improve the results of people with TMDs, which is often confirmed in scientific research [7,24,25,29,53]. It is important to remember that musculoskeletal and ligamentous disorders are not a problem affecting only one region of the body. Usually, the problem is compensated for in another area of the human body, which is why it is so important to have a holistic approach to the problem which the patient reports [4,15,18,38,59].

The studies provided important information on the incidence of cervical spine pain in people with TMDs, but there are limitations that may affect the reception of the tests. Firstly, there is an unequal study and control group, which can mislead readers despite their high statistical significance. The authors did not have the opportunity to exclude people with visual impairments from participating in the study. An additional limitation could be the problem of nocturnal bruxism, which often occurs in patients with TMDs. Additionally, there are reports that nocturnal bruxism will influence changes in cervical tension. Studies of a larger population may provide more reliable results, but due to the time constraints of the study and limited resources, it was not possible to enroll a group of more than 100 people. In addition, it is worth mentioning that each person was examined by one physiotherapist. The inclusion of more specialists in the examination would allow for more efficient testing, but it could differ in the extent to which it is carried out. A significant obstacle may be the narrow age range of people qualified for the study (18–30 years), which is why the authors declare their willingness to continue the above research, paying particular attention to the increase in the number of groups, a wider selection of age groups, and the inclusion of additional variables in the study. In addition, the assessment of pain was limited only to VAS, and enriching the study with other, more extensive scales (for example, the Neck Disability Index) may provide a broader picture of the nature of cervical spine pain. In addition, measurements of the mobility of the cervical spine and temporomandibular joints could be carried out with more advanced tools, but this would entail much higher research costs. In addition, only a few institutions in Poland have such research facilities and employ appropriately trained staff. This study was based on the use of open-source tools that are reliable and common in clinical practice. This study focused on the estimation of the incidence and intensity of cervical spine pain in people with temporomandibular disorders, ignoring aspects of the impact of pain on quality of life. According to the authors, this topic exceeded the scope of the publication and it was decided that it would be the subject of the next report.

## 5. Conclusions

According to population studies, TMDs occur in up to 31% of the population [25] and pain in the cervical spine may affect up to 70% of the population [12]. Disorders within the locomotor system of the masticatory organ are a multifactorial disorder, and patients with this problem are often condemned to concomitant symptoms. The analysis showed that of the 95 people participating in the study, 85.4% reported problems in the cervical spine (*n* = 81), of which almost all people in the study group struggled with this problem (*n* = 43, 97.7%). It is worth adding that in the study group, the level of pain is significantly higher. Moreover, the study results indicate that patients with TMDs are characterized by significantly lower ranges of motion of the temporomandibular joints and the cervical spine compared to the control group. It is important to implement properly planned physiotherapy activities and educate patients in this area. The results of our study suggest that further research is needed to develop the most effective treatment strategies. The obtained research results suggest a clear relationship between the occurrence of TMD and cervical spine pain, but longitudinal studies and other statistical tests are needed to prove this. However, such a broad analysis was not the subject of our study.

## Figures and Tables

**Table 1:** Methodology of the physiotherapeutic examination.

Examination	Description
Palpation [1,3] Standardized starting position: lying/standing position, upper and salt limbs straight, head in neutral position.	
Masseter muscle	Palpation between the zygomatic angle and the angle of the lower jaw.
Temporal muscle	Palpation in 3 parts: - anterior—above the ear and forward from it; - middle—above the ear; - posterior—above the ear and behind the ear.
Medial pterygoid muscle	Palpation on the medial side of the angle of the lower jaw with pressure towards the skull.
Digastric muscle	Palpation in 2 parts during cervical spine extension: - anterior—below the top of the chin on both sides of the floor of the mouth; - posterior—from the back of the angle of the jaw, in front of the sternocleidomastoid muscle towards the ear.
Zygomaticus minor and major muscles	Palpation inside the mouth between the upper lip and the teeth, the other hand performs palpation from the outside.
Suprahyoid and infrahyoid muscles	Gentle palpation during the movement of cervical extension of the spine and maintenance of dental contact.
Sternocleidomastoid muscles	Palpation on the lateral part of the neck, during the movement of lateral flexion of the neck to the opposite side and rotation to the same side.
Anterior/medium/posterior inclined muscles	Palpation on the lateral side of the neck, behind the sternocleidomastoid muscle.
Examination of the range of motion of temporomandibular joints [1,3] Standardized starting position: sitting position, back straight, head in neutral position.	
Abduction—opening the mouth	Active movement measurement: distance between upper and lower incisors.
Protrusion	Active movement measurement: the distance between the upper and lower incisors
Lateral movement (right/left)	Active movement measurement: mandibular protrusion to the left and right, measurement of the distance between the midline of the jaw and the midline of the mandible shifted to the right or left.
Examination of the range of motion of the cervical spine [20] Standardized starting position: sitting position, back straight, head in a horizontal position.	
Flexion and extension	Inclinometer placed on top of the head in position 0°. Movement: flexion and extension (movement in the sagittal plane).
Rotation (Right/Left)	Inclinometer placed on the front of the forehead in position 0°. Movement: rotation to the right and left (movement in the transverse plane).
Lateral flexion (right/left)	Inclinometer placed on top of the head in position 0°. Movement: Right and Left Lateral Flexion (movement in the frontal plane)

**Table 2:** Characteristics of the group.

Characteristics	Control Group *n* = 51	Study Group *n* = 44	Total *n* = 95	*p*-Value
**Gender, *n* (%)**				
Male	13 (25.5)	7 (15.9)	20 (21.1)	0.117
Female	38 (74.5)	37 (84.1)	75 (78.9)	**<0.001**
**Age (years), *n* (%)**				0.085
18–20	16 (31.4)	16 (36.4)	32 (33.7)	
21–25	26 (51.0)	25 (56.8)	51 (53.7)	
26–30	9 (17.6)	3 (6.8)	12 (12.6)	
**Age (mean ± SD)**	22.5 (2.2)	22.2 (3.1)	22.4 (2.7)	0.52
**Health, *n* (%)**				0.076
weak	0 (0.0)	0 (0.0)	0 (0.0)	
sufficient	0 (0.0)	0 (0.0)	0 (0.0)	
good	22 (43.1)	27 (61.4)	49 (51.6)	
very good	20 (39.2)	14 (31.8)	34 (35.8)	
excellent	9 (17.7)	3 (6.8)	12 (12.6)	

Note: SD—standard deviation; statistically significant differences in bold.

**Table 3:** Symptoms of TMDs.

Temporomandibular Disorders—RDC/TMD and a Survey Questionnaire	Control Group *n* = 51	Study Group *n* = 44	Total *n* = 95	ES Cohen’s d	*p*-Value
pain and/or discomfort in the TMJ area, *n* (%)	3 (5.9)	38 (86.4)	41 (43.2)	0.86	**<0.001**
pain in the chewing muscles and/or in the neck muscles, *n* (%)	7 (13.7)	37 (84.1)	44 (46.3)	0.68	**<0.001**
clicking or snapping sound in the TMJ, *n* (%)	7 (13.7)	20 (45.5)	27 (28.4)	0.94	**<0.001**
increased or decreased mobility of the TMJ, *n* (%)	1 (2.0)	17 (38.6)	18 (18.9)	0.86	**<0.001**
rubbing and/or clenching of teeth, *n* (%)	13 (25.5)	25 (56.8)	38 (40.0)	0.94	**<0.001**
numbness in the jaw area, *n* (%)	1 (2.0)	5 (11.4)	6 (6.3)	0.45	0.4328
tinnitus, *n* (%)	12 (23.5)	19 (43.2)	31 (32.6)	0.42	0.099
headaches, *n* (%)	12 (23.5)	42 (95.5)	54 (56.8)	0.94	**<0.001**

Note: TMJ—temporomandibular joint; ES—effect size; statistically significant differences in bold.

**Table 4:** Physiotherapeutic examination (palpation).

Physiotherapy Examination	Control Group *n* = 51	Study Group *n* = 44	Total *n* = 95	ES Cohen’s d	*p*-Value
without facial pain, *n* (%)	40 (78.4)	17 (38.6)	57 (60.0)	0.76	**<0.05**
muscle pain on the left side, *n* (%)	7 (13.7)	19 (43.2)	26 (27.4)	0.68	**<0.05**
pain in the joint on the left side, *n* (%)	1 (2.0)	6 (13.6)	7 (7.4)	0.44	0.081
muscle and joint pain on the left side, *n* (%)	2 (3.9)	10 (22.7)	12 (12.6)	0.57	0.056
muscle pain on the right side, *n* (%)	2 (3.9)	12 (27.3)	14 (14.7)	0.74	**<0.05**
pain in the joint on the right side, *n* (%)	2 (3.9)	5 (11.4)	7 (7.4)	0.28	0.074
muscle and joint pain on the right side, *n* (%)	1 (2.0)	10 (22.7)	11 (11.6)	0.98	**<0.001**

Note: ES—effect size; statistically significant differences in bold.

**Table 5:** Mobility of the temporomandibular joints.

Mobility of the Temporomandibular Joint	Control Group *n* = 51	Study Group *n* = 44	Total *n =* 95	ES Cohen’s d	*p*-Value
Abduction (mean ± SD)	42.3 mm (14.8)	30.9 mm (8.2)	-	0.95	**<0.001**
Pain, *n* (%)	1 (2.0)	19 (43.2)	20 (21.1)		
Protrusion (mean ± SD)	8.2 mm (2.1)	4.6 mm (1.9)	-	0.79	**<0.001**
Pain, *n* (%)	2 (3.9)	27 (61.4)	29 (30.5)		
Lateral movement to the left (mean ± SD)	9.2 mm (1.7)	8.3 mm (1.3)	-	0.59	**<0.001**
Pain, *n* (%)	0 (0.0)	12 (27.3)	12 (12.6)		
Lateral movement to the right (mean ± SD)	9.8 mm (1.1)	7.8 mm (1.8)	-	0.64	**<0.001**
Pain, *n* (%)	1 (2.0)	10 (22.7)	11 (11.6)		

Note: SD—standard deviation; ES—effect size; statistically significant differences in bold.

**Table 6:** Mobility of the cervical spine.

Mobility of the Cervical Spine	Control Group *n* = 51	Study Group *n* = 44	Total *n* = 95	ES Cohen’s d	*p*-Value
Flexion (mean ± SD)	45.6° (12.1)	40.1° (9.9)	-	0.49	**<0.05**
Pain, *n* (%)	1 (2.0)	10 (22.7)	11 (11.6)		
Extension (mean ± SD)	74.1° (5.6)	65.2° (6.3)	-	0.31	0.139
Pain, *n* (%)	1 (2.0)	9 (20.5)	10 (10.5)		
Rotation to the right (mean ± SD)	67.5° (12.7)	59.1° (11.2)	-	0.70	**<0.05**
Pain, *n* (%)	2 (3.9)	8 (18.2)	10 (10.5)		
Rotation to the left (mean ± SD)	68.9° (8.2)	61.7° (10.9)	-	0.75	**<0.05**
Pain, *n* (%)	0 (0.0)	8 (18.2)	8 (8.4)		
Side bend to the right (mean ± SD)	50.7° (8.7)	45.3° (7.9)	-	0.64	**<0.05**
Pain, *n* (%)	3 (5.9)	5 (11.4)	8 (8.4)		
Side bend to the left (mean ± SD)	52.5° (8.5)	45.3° (7.4)	-	0.90	**<0.05**
Pain, *n* (%)	1 (2.0)	6 (13.6)	7 (7.4)		

Note: SD—standard deviation; ES—effect size; statistically significant differences in bold.

**Table 7:** Cervical spine pain and its intensity.

Variable	Control Group *n* = 51	Study Group *n* = 44	Total *n* (%)	ES Cohen’s d	*p*-Value
The cervical spine pain,					
*n* (%)					
Yes	38 (74.5)	43 (97.7)	81 (85.3)	0.85	<0.05 *
No	13 (25.5)	1 (2.3)	14 (14.7)		
Intensity of pain in the cervical spine					
(the VAS Scale), *n* (%)					
0	13 (25.5)	1 (2.3)	14 (14.7)	0.98	<0.001 **
1–3	34 (66.7)	10 (22.7)	44 (46.3)		
4–6	4 (7.8)	20 (45.5)	24 (25.3)		
7–9	0 (0.0)	13 (29.5)	13 (13.7)		
10	0 (0.0)	0 (0.0)	0 (0.0)		

Note: ES—effect size; * chi^2^ = 10.118, df = 1, r_c_ = 0.31; ** chi^2^ = 45.765, df = 4, r_c_ = 0.57, statistically significant differences in bold.

## Data Availability

The data presented in this study are available from the first author upon request.

## References

[1] Osiewicz M.A., Lobbezoo F., Loster B.W., Wilkosz M., Naeije M., Ohrbach R. (2013). Research Diagnostic Criteria for Temporomandibular Disorders (RDC/TMD)—The Polish version of a dual-axis system for the diagnosis of TMD.* RDC/TMD. J. Stoma..

[2] David C.M., Elavarasi P. (2016). Functional anatomy and biomechanics of temporomandibular joint and the far-reaching effects of its disorders. J. Adv. Clin. Res. Insights.

[3] Liem T. (2005). Cranial Osteopathy. Principles and Practice.

[4] Lei J., Liu M.-Q., Jin Yap A.U., Fu K.-Y. (2015). Sleep disturbance and psychologic distress: Prevalence and risk indicators for temporomandibular disorders in a Chinese population. J. Oral Facial Pain Headache.

[5] Velly A.M., Anderson G.C., Look J.O., Riley J.L., Rindal D.B., Johnson K.J., Wang Q., Fricton J., Huff K., Ohrbach R. (2022). Management of painful temporomandibular disorders: Methods and overview of The National Dental Practice-Based Research Network prospective cohort study. J. Am. Dent. Assoc..

[6] Zieliński G., Pająk-Zielińska B., Ginszt M. (2024). A Meta-Analysis of the Global Prevalence of Temporomandibular Disorders. J. Clin. Med..

[7] Crăciun M.D., Geman O., Leuciuc F.V., Holubiac I.S., Gheorghiţă D., Florin F. (2022). Effectiveness of Physiotherapy in the Treatment of Temporomandibular Joint Dysfunction and the Relationship with Cervical Spine. Biomedicines.

[8] De Leeuw R., Bertoli E., Schmidt J.E., Carlson C.R. (2005). Prevalence of traumatic stressors in patients with temporomandibular disorders. J. Oral Maxillofac. Surg..

[9] Manfredini D., Landi L., Bandettini Di Poggio A., Dell’Osso L., Bosco M. (2003). A critical review on the importance of psychological factors in temporomandibular disorders. Minerva Stomatol..

[10] Jussila P., Knuutila J., Salmela S., Näpänkangas R., Päkkilä J., Pirttinirmi P., Raustia A. (2018). Association of risk factors with temporomandibular disorders in the Northern Finland Birth Cohort 1966. Acta Odontol. Scand..

[11] Brancher J.A., Spada P.P., Meger M.N., Fatturi A.L., Dalledone M., de Paiva Bertoli F.M., Deeley K., Scariot R., Rezende Viera A., Calvano Küchler E. (2019). The association of genetic polymorphisms in serotonin transporter and catechol-O-methyltransferase on temporomandibular disorders and anxiety in adolescents. J. Oral Rehabil..

[12] Berger M., Szalewski L., Bakalczuk M., Bakalczuk G., Bakalczuk S., Szkutnik J. (2015). Association between estrogen levels and temporomandibular disorders: A systematic literature review. Menopause Rev..

[13] Kapos F.P., Exposto F.G., Oyarzo J.F., Durham J. (2020). Temporomandibular disorders: A review of current concepts in aetiology, diagnosis and management. Oral Surg..

[14] Kazeminasab S., Nejadghaderi S.A., Amiri P., Pourfathi H., Araj-Khodaei M., Sullman M.M.S., Kolahi A.-A., Safiri S. (2022). Neck pain: Global epidemiology, trends and risk factors. BMC Musculoskelet. Disord..

[15] Oleksy Ł., Kielnar R., Mika A., Jankowicz-Szymańska A., Bylina D., Sołtan J., Pruszczyński B., Stolarczyk A., Królikowska A. (2021). Impact of Cervical Spine Rehabilitation on Temporomandibular Joint Functioning in Patients with Idiopathic Neck Pain. Biomed Res. Int..

[16] Nunes A.M., Rosario Lopes P.R., Viera Bittencourt M.A., de Araujo R.P.C. (2020). Association between severity of the temporomandibular disorder, neck pain, and mandibular function impairment. Rev. CEFAC.

[17] Kim D., Ko S.-G., Lee E.-K., Jung B. (2019). The relationship between spinal pain and temporomandibular joint disorders in Korea: A nationwide propensity score-matched study. BMC Musculoskelet. Disord..

[18] De Boever J.A., Nilner M., Orthlieb J.-D., Steenks M.H. (2008). Recommendations by the EACD for examination, diagnosis, and management of patients with temporomandibular disorders and orofacial pain by the general dental practitioner. J. Orofac. Pain.

[19] Reed M.D., Nostran W.V. (2014). Assessing pain intensity with the visual analog scale: A plea for uniformity. J. Clin. Pharmacol..

[20] Jordan K. (2000). Assessment of published reliability studies for cervical spine range-of-motion measurement tools. J. Manip. Physiol. Ther..

[21] Manfredini D., Arveda N., Guarda-Nardini L., Segù M., Collesano V. (2012). Distribution of diagnoses in a population of patients with temporomandibular disorders. Oral Surg. Oral Med. Oral Pathol. Oral Radiol..

[22] Rodrigues Conti P.C., Pinto-Fiamengui L.M.S., Ortigosa Cunha C., de Castro Ferreira Conti A.C. (2012). Orofacial pain and temporomandibular disorders: The impact on oral health and quality of life. Braz. Oral Res..

[23] Zakrzewska J.M. (2015). Temporomandibular disorders, headaches and chronic pain. J. Pain Palliat. Care Pharmacother..

[24] Al-Jundi M.A., John M.T., Setz J.M., Szentpétery A., Kuss O. (2008). Meta-analysis of treatment need for temporomandibular disorders in adult nonpatients. J. Orofac. Pain..

[25] Li D.T.S., Leung Y.Y. (2021). Temporomandibular Disorders: Current Concepts and Controversies in Diagnosis and Management. Diagnostics.

[26] Loster J.E., Osiewicz M.A., Groch M., Ryniewicz W., Wieczorek A. (2017). The Prevalence of TMD in Polish Young Adults. J. Prosthodont..

[27] Manfredini D., Guarda-Nardini L., Winocur E., Piccotti F., Ahlberh J., Lobbezzo F. (2011). Research diagnostic criteria for temporomandibular disorders: A systematic review of axis I epidemiologic findings. Oral Surg. Oral Med. Oral Pathol. Oral Radiol. Endod..

[28] Manfredini D., Piccotti F., Ferronato G., Guarda-Nardini L. (2010). Age peaks of different RDC/TMD diagnoses in a patient population. J. Dent..

[29] Liu F., Steinkeler A. (2013). Epidemiology, diagnosis, and treatment of temporomandibular disorders. Dent. Clin. N. Am..

[30] Walczyńska-Dragon K., Baron S., Nitecka-Buchta A., Tkacz E. (2014). Correlation between TMD and cervical spine pain and mobility: Is the whole body balance TMJ related?. Biomed. Res. Int..

[31] Torres F., Campos L.G., Fillipini H.F., Weigert K.L., Vecchia G.F.D. (2012). Efeitos dos tratamentos fisioterapêutico e odontológico em pacientes com disfunção temporomandibular. Fisioter. Mov..

[32] Wolan-Nieroda A., Maciejczak A., Mańko G., Juszczyk K., Rutkowski S., Guzik A. (2023). Comparative Analysis of Mandibular and Cervical Mobility in Young Adults with Temporomandibular Joint Disorders: A Case-Control Study. Med. Sci. Monit..

[33] Bonjardim L.R., Gavião M.B.D., Pereira L.J., Castelo P.M., Garcia R.C.M.R. (2005). Signs and symptoms of temporomandibular disorders in adolescents. Braz. Oral Res..

[34] Yap A.U., Chen C., Wong H.C., Yow M., Tan E. (2021). Temporomandibular disorders in prospective orthodontic patients. Angle Orthod..

[35] Colonna A., Guarda-Nardini L., Ferrari M., Manfredini D. (2021). COVID-19 pandemic and the psyche, bruxism, temporomandibular disorders triangle. Cranio.

[36] Gonçalves D., Bigal M.E., Jales L.C.F., Camparis C.M., Speciali J.G. (2010). Headache and symptoms of temporomandibular disorder: An epidemiological study. Headache.

[37] Troeltzsch M., Troeltzsch M., Cronin R.J., Brodine A.H., Frankenberger R., Messlinger K. (2011). Prevalence and association of headaches, temporomandibular joint disorders, and occlusal interferences. J. Prosthet. Dent..

[38] Krzyżanowski M., Hansdorfer-Korzon R., Rajkowska-Labon E. (2014). The physiotherapeutic estimation of the act of temporal-mandibular joints among students of Medical University of Gdańsk. Fizjoterapia Pol..

[39] Exposto F.G., Renner N., Bendixen K.H., Svensson P. (2021). Pain in the temple? Headache, muscle pain or both: A retrospective analysis. Cephalalgia.

[40] Yeler D.Y., Yılmaz N., Koraltan M., Aydın E. (2017). A survey on the potential relationships between TMD, possible sleep bruxism, unilateral chewing, and occlusal factors in Turkish university students. Cranio.

[41] Johansson A., Unell L., Carlsson G.E., Söderfeldt B., Halling A. (2003). Gender difference in symptoms related to temporomandibular disorders in a population of 50-year-old subjects. J. Orofac. Pain.

[42] Çebi A.T. (2023). Presence of tinnitus and tinnitus-related hearing loss in temporomandibular disorders. Cranio.

[43] Mottaghi A., Menéndez-Díaz I., Cobo J.L., González-Serrano J., Cobom T. (2019). Is there a higher prevalence of tinnitus in patients with temporomandibular disorders? A systematic review and meta-analysis. J. Oral Rehabil..

[44] Skog C., Fjellner J., Ekberg E.C., Häggman-Henriksonm B. (2019). Tinnitus as a comorbidity to temporomandibular disorders—A systematic review. J. Oral Rehabil..

[45] Hassel A.J., Rammelsberg P., Schmitter M. (2006). Inter-examiner reliability in the clinical examination of temporomandibular disorders: Influence of age. Community Dent. Oral Epidemiol..

[46] Kitsoulis P., Marini A., Iliou K., Galani V., Zimpis A., Kanavaros P., Paraskevas G. (2011). Signs and symptoms of temporomandibular joint disorders related to the degree of mouth opening and hearing loss. BMC Ear Nose Throat Disord..

[47] Ferreira M.P., Waisberg C.B., Conti P.C.R., Bevilaqua-Grossi D. (2019). Mobility of the upper cervical spine and muscle performance of the deep flexors in women with temporomandibular disorders. J. Oral Rehabil..

[48] De Laat A., Meuleman H., Stevens A., Verbeke G. (1998). Correlation between cervical spine and temporomandibular disorders. Clin. Oral Investig..

[49] Lendraitiene E., Smilgiene L., Petruseviciene D., Savickas R. (2021). Changes and Associations between Cervical Range of Motion, Pain, Temporomandibular Joint Range of Motion and Quality of Life in Individuals with Migraine Applying Physiotherapy: A Pilot Study. Medicina.

[50] Minervini G., Franco R., Marrapodi M.M., Crimi S., Badnjević A., Cervino G., Bianchi A., Ciciu M. (2023). Correlation between Temporomandibular Disorders (TMD) and Posture Evaluated trough the Diagnostic Criteria for Temporomandibular Disorders (DC/TMD): A Systematic Review with Meta-Analysis. J. Clin. Med..

[51] Greenbaum T., Dvir Z., Emodi-Perlman A., Reiter S., Rubin P., Winocur E. (2021). The association between specific temporomandibular disorders and cervicogenic headache. Musculoskelet. Sci. Pract..

[52] Cortese S., Mondello A., Galarza R., Biondi A. (2017). Postural alterations as a risk factor for temporomandibular disorders. Acta Odontol Latinoam..

[53] Kielnar R., Mika A., Bylina D., Sołtan J., Stolarczyk A., Pruszczyński B., Recheniuk H., Szczegielniak J., Królikowska A., Oleksy Ł. (2021). The influence of cervical spine rehabilitation on bioelectrical activity (sEMG) of cervical and masticatory system muscles. PLoS ONE.

[54] Cuccia A., Caradonna C. (2009). The relationship between the stomatognathic system and body posture. Clinics.

[55] Weber P., Rodrigues Corrêa E.C., dos Santos Ferreira F., Corrêa Soares J., de Paula Bolzan G., da Silva A.M.T. (2012). Cervical spine dysfunction signs and symptoms in individuals with temporomandibular disorder. J. Soc. Bras. Fonoaudiol..

[56] Raya C.R., Plaza-Manzano G., Pecos-Martin D., Ferragut-Garcias A., Martin-Casas P., Gallego-Izquierdo T., Romero-Franco N. (2017). Role of upper cervical spine in temporomandibular disorders. J. Back Musculoskelet. Rehabil..

[57] Munhoz W.C., Marques A.P., Tesseroli de Siqueira T.T. (2005). Evaluation of body posture in individuals with internal temporomandibular joint derangement. Cranio.

[58] Pedroni C.R., de Oliveira A.S., Berzin F. (2006). Pain characteristics of temporomandibular disorder—A pilot study in patients with cervical spine dysfunction. J. Appl. Oral Sci..

[59] Manfredini D., Castroflorio T., Perinetti G., Guarda-Nardini L. (2012). Dental occlusion, body posture and temporomandibular disorders: Where we are now and where we are heading for. J. Oral Rehabil..

[60] Zieliński G., Matysik-Woźniak A., Baszczowski M., Rapa M., Ginszt M., Pająk B., Szkutnik J., Rejdak R., Gawda P. (2023). Myopia & painful muscle form of temporomandibular disorders: Connections between vision, masticatory and cervical muscles activity and sensitivity and sleep quality. Sci. Rep..

[61] Kang J.-H. (2020). Effects on migraine, neck pain, and head and neck posture, of temporomandibular disorder treatment: Study of a retrospective cohort. Arch. Oral Biol..

